# Modification of Mechanical Properties of Aluminum Alloy Rods via Friction-Extrusion Method

**DOI:** 10.3390/ma13225224

**Published:** 2020-11-19

**Authors:** Anna Wójcicka, Krzysztof Mroczka, Jerzy Morgiel

**Affiliations:** 1 Institute of Technology, Pedagogical University of Cracow, 30-084 Kraków, Poland; krzysztof.mroczka@up.krakow.pl; 2 Institute of Metallurgy and Material Science, Polish Academy of Sciences, 30-059 Kraków, Poland; j.morgiel@imim.pl

**Keywords:** friction extrusion, gradient microstructure, aluminum alloy, wire

## Abstract

The elaboration of a modified friction-extrusion method aimed at obtaining 2017A aluminum rods of gradient microstructure is described. This was achieved by cutting spiral grooves on the face of the stamp used for alloy extrusion. The experiments were carried out at a constant material feed (~10 mm/min) and a range of tool rotation speeds (80 to 315 rpm). The microstructure observations were carried out using light microscopy (LM) and both scanning and transmission electron microscopy (SEM and TEM). The mechanical properties were assessed through hardness measurements and static tensile tests. The performed investigations show that material simultaneous radial and longitudinal flow, enforced by friction of the rotating tool head and extrusion, results in the formation of two zones of very different microstructures. At the perpendicular section, the outer zone stands out from the core due to circumferential elongation of strings of particles, while in the inner zone the particles are arranged in a more uniform way. Simultaneously, the grain size of the outer zone is refined by two to four times as compared with the inner one. The transfer from the outer zone to the core area is of gradient type. The hardness of the outer zone was found to be ~10% to ~20% higher than that of the core.

## 1. Introduction

Plastic deformation (PD) of metals and alloys—realized in a number of ways—can be very effective in controlling the shape, microstructure and mechanical properties of the final products. Some limitations of this approach were found to be a low number of easy crystallographic slip systems in some metallic materials, or their unfavorable shape, such as in the case of aluminum scrap. A solution to the latter problem was found in merging friction stirring (FS) and extrusion (E) processes [1,2,3,4,5]. The complex mechanical loading conditions arising during the application of this solution allowed for the deformation paths plasticizing aluminum machining chips to be continuously changed and for the chips to be transformed into a solid fine-grain wire [4,5].

Coupling individual PD processes into one may be used not only to form hard-to-work materials, but also to produce parts of predetermined gradient microstructure. The approach borrowed from friction steer welding (FSW) [6,7,8] is especially useful in such applications as it is capable of locally varied strong microstructure refinement. Previous experiments used this idea in such a way that spiral grooves covered all of the face of an extrusion die [4,5]. The above approach allowed the obtainment of a material characterized by grain size in the near micrometer range and spirally distributed refined crushed precipitates mixed together with contaminant oxide particles. The ease of joining E and FS processes offers the possibility of experimenting with them, especially as concerns the parts controlling the flow of the worked material, i.e., the plunger face. The gradient structure is created in various metallic materials to increase their strength [9,10,11] or obtain better electrical properties [12]. In most cases, obtaining this microstructure in a wire requires a multi-stage process based on plastic deformation by twisting [12] or compression bonding [11].

The present work is aimed at testing the effectiveness of a newly developed hollow plunger with a spiral cut on its face and an opening in the center for the friction stir extrusion (FSE) processing of 2017A aluminum alloy. Incision of grooves made only at the external rim activates radial flow of the processed material, differentiating the degree of deformation at the outside and inside of the received rod-like product. The resulting microstructure was analyzed with light microscopy (LM) as well as both scanning and transmission electron microscopy (SEM/TEM), while mechanical properties were assessed by tensile tests and hardness measurements. The tested method could be applied for the production of aluminum wires o improved conductivity to strength ratio. Alternatively, it could be used for particle refining at the outer layer of metal matrix composite rods, increasing their wear resistance.

## 2. Experimental Procedure

The extruded 2017A aluminum alloy (marked as E) wire rod as well as re-melted castings (marked as RM) from the same alloy (serving as a model material) were used in the experiment. The chemical composition of this material is as follows: 4.14% Cu, 0.72% Mg, 0.6% Mn, 0.68% Si, 0.18% Zn, 0.31% Fe, balance Al, in wt %.

**Figure 1:**
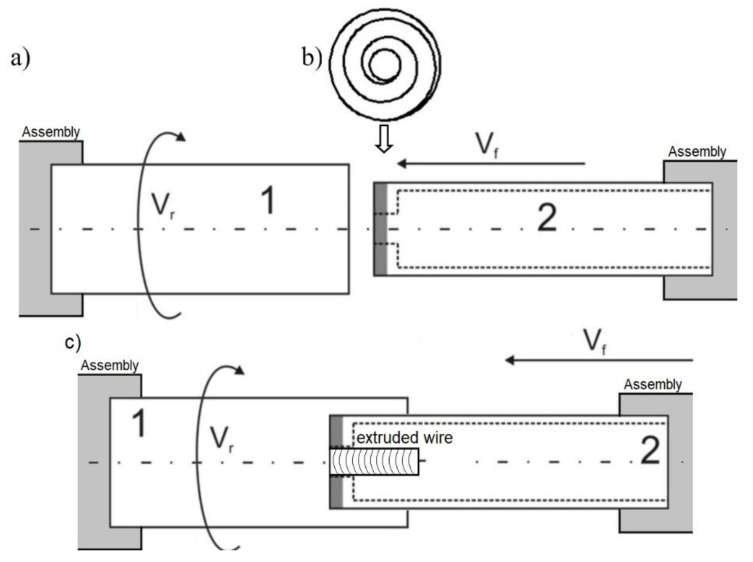
Scheme of the elaborated friction-extrusion experiment: (**a**) arrangement of the piece of the worked material (1) in relation to stamp (2) pressed into it, (**b**) spiral groove cut at the stamp face (shaded with gray) (**c**) during the process.

**Figure 2:**
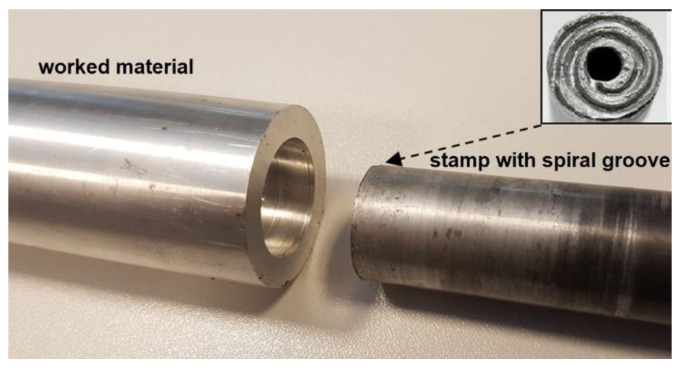
Image of the stand with the processed material removed (inset presents the grooves cut at the stamp face).

The friction-extrusion experiment was executed by placing the worked piece of aluminum alloy in a cylinder, rotating at speeds from 80 to 315 rpm/min. Next, the stamp (of 18 mm diameter), with a spiral groove at its face and an opening in its center, was pressed into it at a rate of ~10 mm/min, as schematically shown in Figure 1. An image of the stand with the processed material removed is shown in Figure 2.

The microstructure observations were performed using light microscopy (Olympus GX51, Tokio, Shinjuku, Japan), scanning electron microscopy (Philips M525, Eindhoven, The Netherlands), and transmission electron microscopy (Tecnai FEG 200 kV, Eindhoven, Holland) methods. The specimens for LM were polished and etched in a solution containing 2 mL HF, 4 mL HNO_3_ and 94 mL H_2_O. The hardness was measured with a Vickers indenter under 100 g load and spacing of ~0.5 mm.

## 3. Results

**Figure 3:**
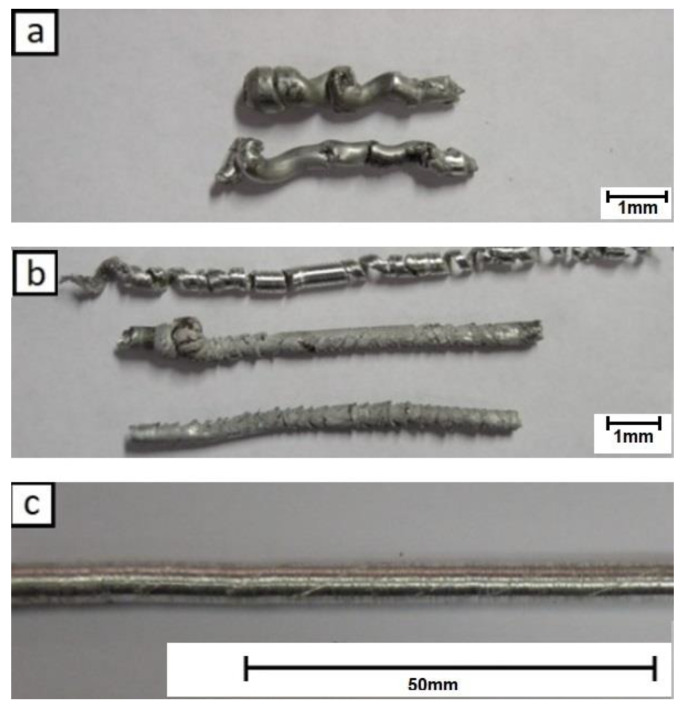
Extrusion experiments with re-melted castings (RM) alloy: (**a**) 80 rpm, (**b**) 160 rpm and (**c**) 224 rpm (all presented at the same scale).

The preliminary friction extrusion tests showed that only those performed at higher rotation speeds allowed the obtainment of relatively straight rods free from major surface defects. The experiments run at medium to low speed gave only twisted short pieces with a corrugated surface (Figure 3).

**Figure 4:**
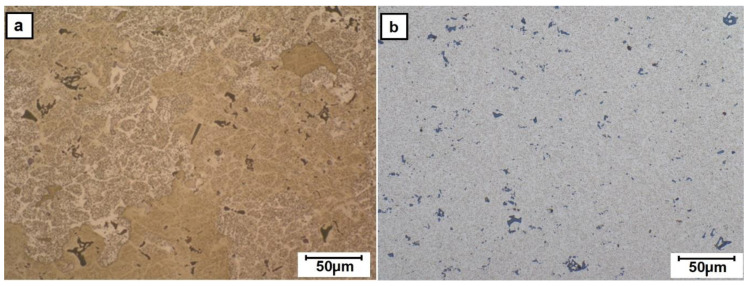
Light microscopy (LM) images of microstructure of sections of 2017A rods prepared for friction-extrusion test of: (**a**) RM and (**b**) extrusion (E) alloy (as purchased).

**Figure 5:**
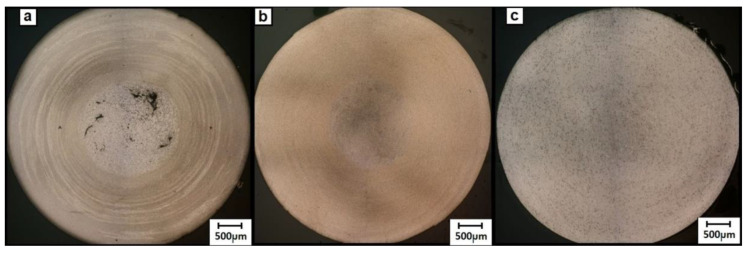
LM images of cross-sections of friction-extruded alloys processed at various rotations: (**a**) RM/224 rpm, (**b**) E/224 rpm and (**c**) E/315 rpm.

**Figure 6:**
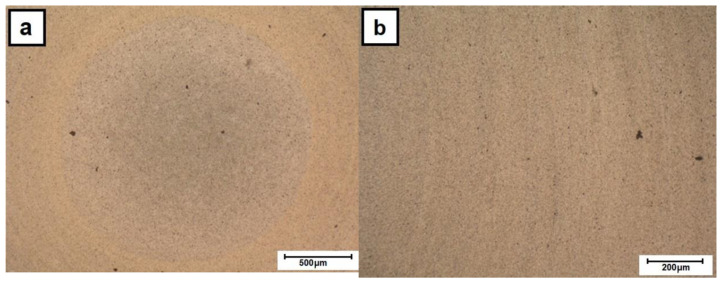
LM images of cross-sections of friction-extruded E alloy at 224 rpm: (**a**) core and (**b**) outer-zone area.

The light microscopy investigation of the cross-section of as-cast 2017A rod indicated that it is characterized by the presence of numerous phases as evidenced by their darker and lighter contrast (Figure 4a). The as-purchased extruded material still shows the presence of precipitates but generally of smaller size and more uniform distribution (Figure 4b). Subjecting the cast alloy to friction-extrusion processing caused some refining of the microstructure in the core area of the rod, but the porosity formed during solidification was mostly retained (Figure 5a). This area strongly differs from the clearly cut outer rim, whose microstructure was much more refined and carried constituent phases in radially arranged thin strings. The observations with the same method but at higher magnification revealed that the transition from one area to the other is a gradual one and the continuity of the material is always preserved (Figure 6). Simultaneously, the experiment showed that the ratio of the rim to core material remained the same within the whole applied rotation speed of the punch.

**Figure 7:**
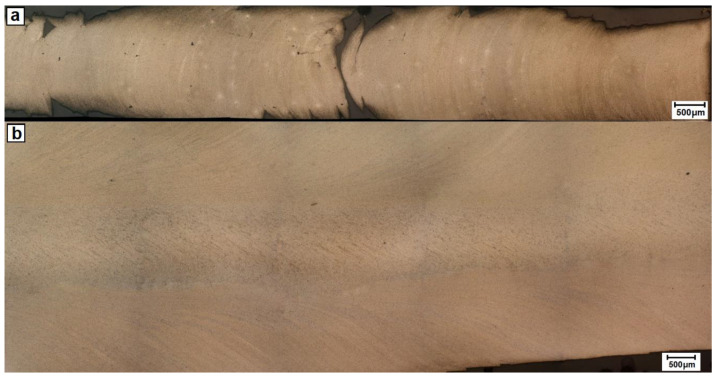
LM images of longitudinal section of E/224 rpm cut at: (**a**) ~0.2 mm from surface and (**b**) through its center.

The polishing of the side of the rod (down to ~0.2 mm) revealed both significant surface roughness and evident bending of the radial strings, this being a direct proof of the simultaneous radial and longitudinal flow of the processed alloy (Figure 7a). The section made along the rod center (i.e., at a distance of ~2.3 mm from the surface) showed that the bending of the lines documenting the flow of the material in the outer ring is higher when closer to the core area, i.e., eventually aligning itself along the rod long axis (Figure 7b). The detailed examination of the core area revealed that it also contained a set of fine bends arranged at an angle to the stamp axis.

**Figure 8:**
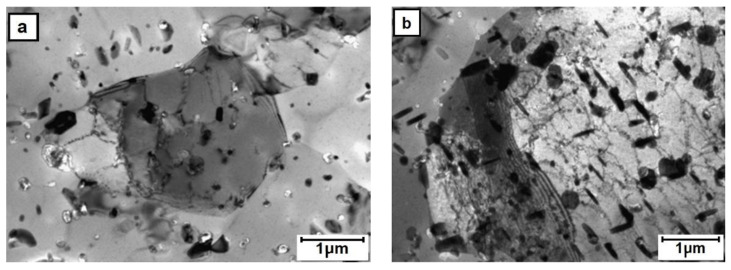
TEM microstructure of the core area (E/224 rpm) presenting: (**a**) fine and (**b**) coarse grain areas.

**Figure 9:**
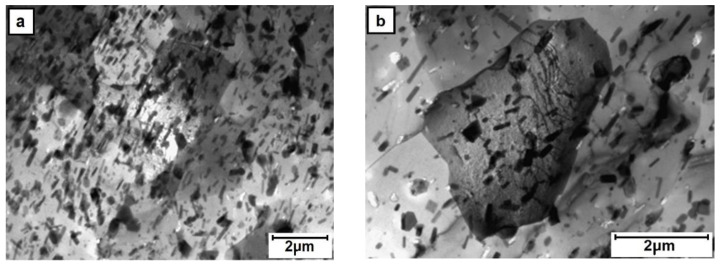
TEM microstructure of the outer-zone area (E/224 rpm) presenting presence of: (**a**) extra-fine and (**b**) fine grain areas.

The TEM observations of thin foils prepared from the perpendicular section of the core area processed alloy showed that it is filled with roughly equiaxed grains strongly differing in size, i.e., from ten to several tens of microns (Figure 8). The grains and their boundaries were filed with rod-like or plate-like precipitates of sizes up to 5 μm. The presence of dislocations anchored at precipitates and occasionally arranged in polygonal walls was also noted. The TEM observation of the outer-rim area helped to establish that even as the grains are also mostly equiaxed, they are much smaller than those in the core, i.e., of a grain size between 2 and 10 μm (Figure 9). The precipitates in this area were smaller, but their density was higher than that in the core area. Only the dislocation density and their arrangement were comparable.

**Figure 10:**
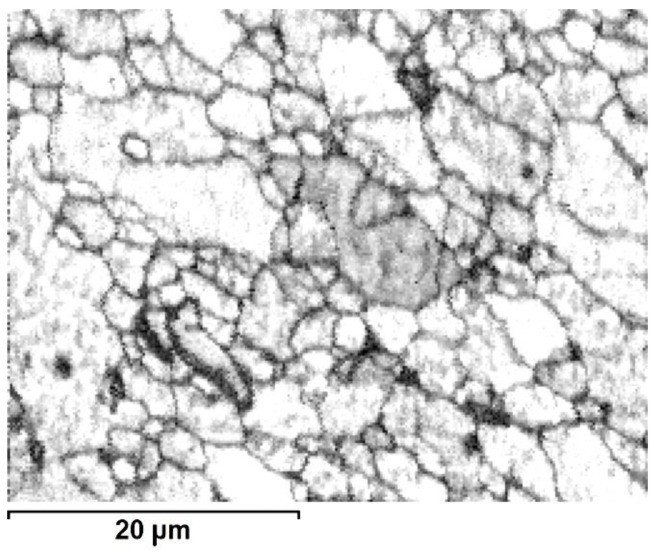
Image based on quality ratio of electron diffraction pattern (SEM/EBSD) from core area (E/224 rpm).

**Figure 11:**
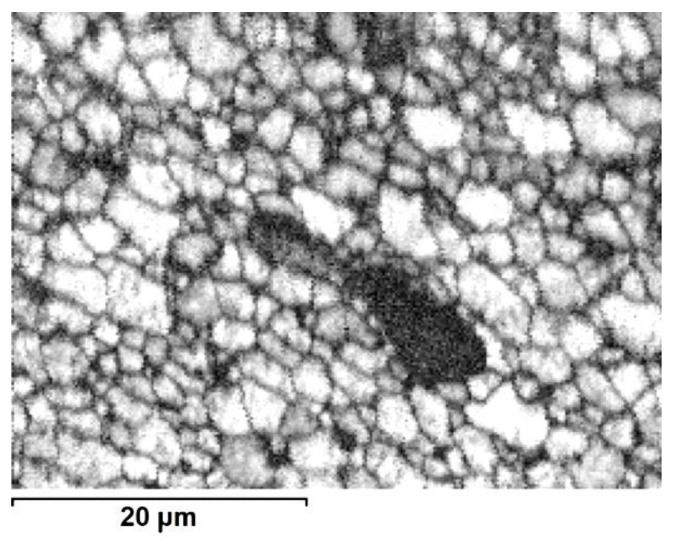
Image based on quality ratio of electron diffraction pattern (SEM/EBSD) from core/outer-zone area boundary (E/224 rpm).

**Figure 12:**
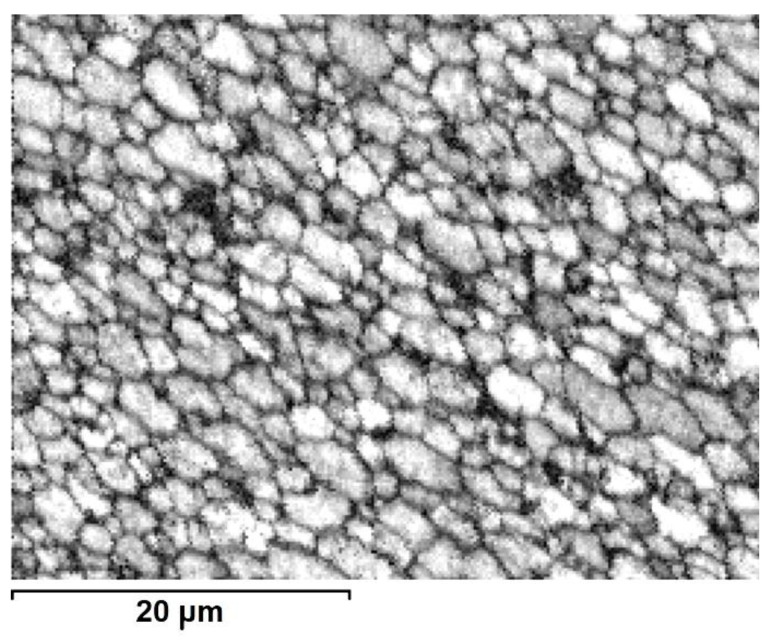
Image based on quality ratio of electron diffraction pattern (SEM/EBSD) from outer-zone area (E/224 rpm).

The SEM electron backscatter diffraction EBSD images obtained using the quality ratio of acquired electron diffraction of the 2017A alloy confirmed that the core area is on a par filled by large grains and interspersed groups of smaller ones (Figure 10). The same type of measurements performed at the boundary between the core and outer-zone area show only the occasional presence of large grains immersed in a fine-grain matrix (Figure 11), while the outer-zone area is characterized by a uniform, fine microstructure (Figure 12).

**Figure 13:**
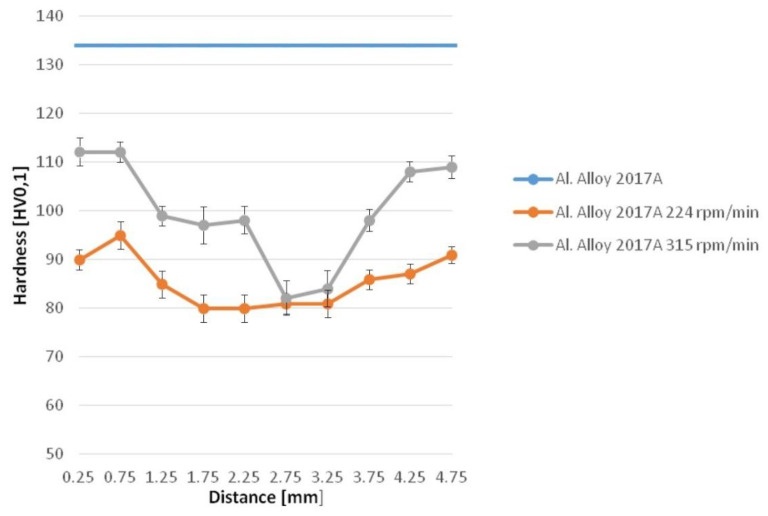
Hardness profiles measured on section of E and friction-extruded rods.

The mechanical properties of friction-extruded material were assessed by hardness and static tensile tests. The hardness measurements along the cross-section of 2017A rod showed that at all rotation speeds the core area is usually 10–20% softer than the outer zone (Figure 13). Simultaneously, at the faster applied rotations, the average hardness was higher in respective areas. It is notable that the hardness measured across a section of a reference rod (as-fabricated/extruded) was also higher than any part of the friction-extruded rods.

**Figure 14:**
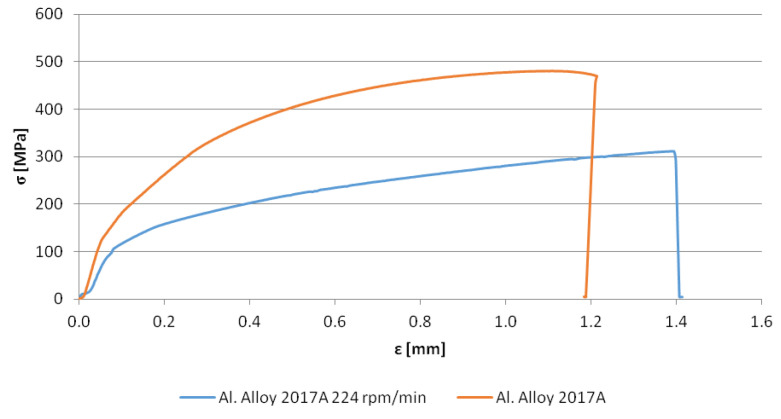
Static tensile test of the rod received after 224 rpm of the punch and a reference one.

The static tensile test of the rods obtained through this process gave reproducible results only for those received after 224 rpm of the punch (lower and higher speeds produced a specimen of defected geometry or notched surface, respectively). The yield strength (R_p0.2_) determined based on the graph was close to 100 MPa, while ultimate tensile strength UTS was slightly above 300 MPa (Figure 14). Both these values were lower than that proper for the reference material, except the slightly larger (~10%) plastic deformation range.

**Figure 15:**
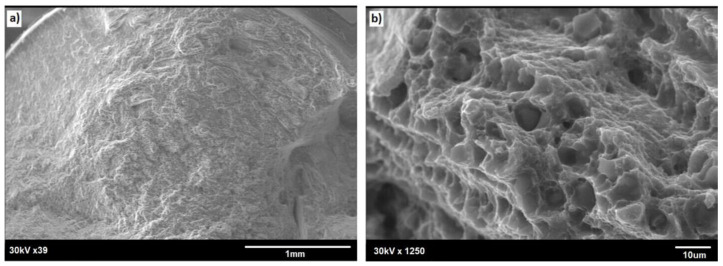
SEM image of fracture surface of E/224 rpm rod: (**a**) general view and (**b**) core area.

**Figure 16:**
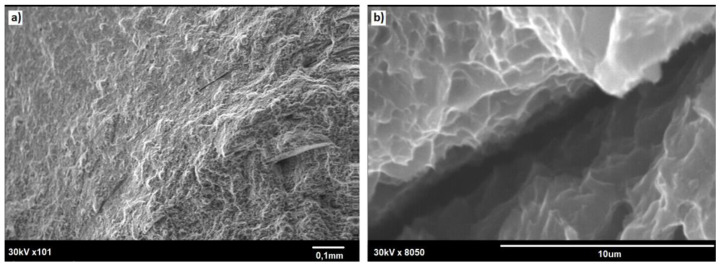
SEM image of fracture surface of E/224 rpm rod: (**a**) outer zone/core boundary and (**b**) crack marked with an arrow in (**a**).

**Figure 17:**
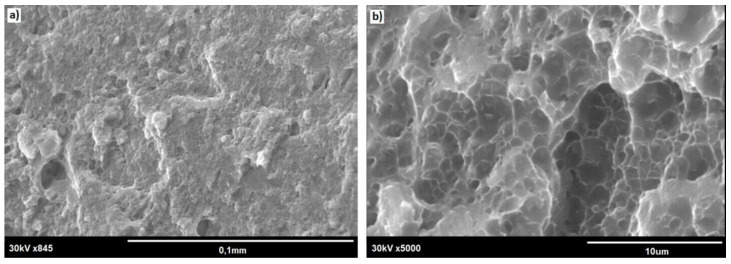
SEM image of fracture of E/224 rpm rod: view of the outer-zone area. (**a**) outer rim; (**b**) core area.

The SEM observation of the fractured specimens revealed that cracking tended to start at the small notches present at the rod surface (Figure 15a). Both the outer rim and the core showed dimples, these being characteristic features of plastic fracture (compare Figure 15 and Figure 16). Simultaneously, the core area was always slightly raised, documenting its more plastic properties. Some of the tensile tested specimens also showed the presence of deep cracks delineating the boundary between the outer rim and the core area (as shown by solid arrows in Figure 17), but these seemed to have no effect on the fracture mode of the rod.

## 4. Discussion

The presently elaborated version of friction stir extrusion (FSE) processing of malleable alloys, such as aluminum alloys, is based on a novel idea of simultaneous merging of axial and radial flow of the material. Pressing the punch with a spiral groove cut at the outside of its face and a hole at the center allows the obtainment of rods, whose outer zone is the subject of a much higher level of plastic deformation than that of the core. The above was documented using a model cast aluminum alloy with a number of large precipitates, which were crushed and served as deformation path markers. The heat release accompanying FSE processing stimulates the dynamic recovery of the crystal structure, resulting in refined grain size being higher for the more worked material. In effect, in one pass, one receives a rod of unique microstructure characterized by a fine-grain outer zone and a coarser grain core. The previous attempts to merge friction with extrusion were limited to that with the spiral grooves covering all of the punch face [13]. It also allowed us to obtain fine-grain material but of approximately the same size for the whole processed volume.

The LM observations on the cross and axial sections of the presented friction-extruded rods showed that the material in the outer rim is the subject of both radial and axial flow. The latter gains significance at the approach to the boundary zone with the core area, at which the flow line is arranged practically along the extrusion direction, i.e., the spiral paths are gradually straightened to longitudinal ones. The boundary zone is filled with the most highly refined grains dissolving any flow lines, which however, re-emerge in the core and are parallel to those in the center of the bottom of the outer zone. The above gave a close resemblance to the microstructure of friction stir welded plates with the most refined microstructures between their side and the so-called “nugget” left behind the pushing-ahead rotating tool [1,6]. In the present case, the role of the rotating pin is taken over by the grooved rim of the punch, but a close similarity of its microstructure to that obtained in FSW processing is evident.

Hardness measurements acquired from sections of the rods obtained with this method tend to give a saddle-like profile centered on the rod’s long axis. These results are practically wholly dependent on local grain size as in the case of FSW processes [14,15]. This phenomenon could be explained according to the Hall–Petch relationship, i.e., in the same way as in the case of 1050 and 5083 Al alloys [16]. On the other hand, during such processing, the material temperature is raised high enough to cause its over-ageing and resulting softening as compared to that of as-fabricated extruded reference rods. Simultaneously, increasing the punch rotation speed from ~200 to ~300 rpm/min raised the hardness of the outer part by ~20% but not the center of the core. The above is proof that it is the outer rim that receives most of the radial flow of the material, which is characterized by finer grains the faster it rotates. Simultaneously, the core receives mostly the axially flown material controlled by the punch liner speed, which is kept constant throughout these experiments, i.e., its hardness remained the same for samples received in all trials.

Examination of the fractured surfaces of the rods subjected to tensile tests confirmed that their UTS was very sensitive to side-surface roughness (micro-notches) serving as crack propagation centers. The above suggests that the production of stronger rods with the FE method will require either polishing of their surface or even better optimization of the punch longitudinal and rotation speeds. These observations also revealed the presence of occasional discontinuity between the outer zone and the core material, which—under loading during tensile tests—transforms into deep circumferential openings. The discontinuity between material subjected to differing flow rates was previously noted between the FSW “nugget” and the welded plates [17], but adjustment of the processing parameters helped to practically eliminate its presence, opening way for even the most demanding applications. Further work on material production with the tested method will also be aimed at eliminating such discontinuities.

## 5. Summary

The modified friction-extrusion method, based on pushing a spirally grooved rotating piston with an opening in the center, helped to produce a rod of strongly changing microstructure and properties. Its outer zone was composed of fine grains formed from the material, which reached that place on longer spiral paths and were the subject of high plastic deformation. The core area was filled with coarser grains, also being a result of dynamic recovery, but of material that was nearly axially pushed through the central opening in the punch and therefore underwent less plastic deformation. The decrease in hardness as compared with the reference material indicates that the friction-extrusion resulted in its over-ageing. Therefore, the saddle-like hardness profile should be directly tied to changing grain size according to the Hall–Petch relation. Finally, the presently proposed method opens the way for one-step fabrication of rods or wires of radially changing microstructure.
